# Antipsychotic prescribing trends in England: role of regional disparities, health inequalities and ethnic density

**DOI:** 10.1192/bjo.2026.10989

**Published:** 2026-02-23

**Authors:** Muhammad Umair Khan, Syed Shahzad Hasan, Ian Maidment, Nusrat Husain

**Affiliations:** Aston Pharmacy School, College of Health and Life Sciences, Aston University, UK; School of Applied Sciences, University of Huddersfield, UK; School of Health Sciences, Division of Psychology and Mental Health, The University of Manchester, UK; Mersey Care NHS Foundation Trust, Prescot, UK

**Keywords:** Antipsychotic, ethnic density, health inequalities, England, psychosis

## Abstract

**Background:**

Antipsychotics are essential for managing certain mental disorders; however, little is known about regional disparities in their prescribing or how these patterns are shaped by ethnic density and health inequalities.

**Aims:**

To analyse national, regional and local integrated care board trends in antipsychotic prescribing in England from April 2019 to March 2025, and to explore their associations with health inequalities and ethnic density.

**Method:**

A population-level observational study was conducted using the English primary care prescription data from OpenPrescribing. Linear regression was used to assess trends in first-generation (FGA), second-generation (SGA) and total antipsychotic prescribing. Generalised additive models examined associations between prescription rates and health inequalities and ethnic density at the local level.

**Results:**

Antipsychotic prescribing increased from 185.55 to 199.85 prescriptions per 1000 population between April 2019 and March 2025. SGA use increased significantly (168.48 to 186.27) whereas FGA use declined (17.08 to 13.58). Regional annual increases ranged from 3.85% (95% CI = 3.53%, 4.16%) in London to −0.21% (95% CI = −0.72%, 0.31%) in the South-West region, with greater variation at the local level, from 6.62% (95% CI = 5.71%, 7.53%) in North Central London to −2.05% (95% CI = −2.71%, −1.40%) in Shropshire, Telford and the Wrekin. Higher Pakistani ethnic density was associated with lower prescribing rates, whereas greater health inequalities were linked to increased prescribing.

**Conclusions:**

Antipsychotic prescribing patterns have shifted in recent years, with notable regional disparities influenced by health inequalities and ethnic composition. Targeted interventions are needed to promote equitable access and address prescribing disparities in mental healthcare.

Mental disorders are one of the leading causes of disease burden worldwide. The Global Burden of Disease study reported that, in 2019, approximately 970 million people were living with mental disorders, resulting in 125 million disability-adjusted life years (DALYs) (GBD 2022).^1^ In 2022 a study reported 418 million DALYs, with an economic loss of USD 5 trillion.^2^ In England, recent National Health Service (NHS) data show that 1 in 6 adults experienced a mental condition in 2022/23, with 3.58 million people accessing NHS services at a cost to the NHS of £16 billion.^3^

Antipsychotics play a crucial role in managing mental disorders such as schizophrenia, bipolar disorder and severe depression. They are traditionally categorised into first-generation antipsychotics (FGAs) and second-generation antipsychotics (SGAs), although newer classification frameworks have proposed a distinct group of third-generation agents such as dopamine partial agonists.^4^ Both FGAs and SGAs have proven effective in managing mental disorders.^5^ Whereas SGAs are often less associated with extrapyramidal symptoms compared with FGAs, they have been linked to other adverse effects such as weight gain and metabolic disorders.^6,7^ The risks associated with all antipsychotics render the monitoring of prescribing trends a significant public health issue.

Global trends have shown an overall increase in antipsychotic use, particularly SGAs.^8^ In the UK, studies have reported a consistent increase in antipsychotic prescriptions in conditions such as non-affective psychosis, anxiety disorders, depression and conduct disorders, with SGAs accounting for a majority of the prescriptions.^9–14^ Although these trends were well established before 2020, the COVID-19 pandemic introduced new challenges to mental healthcare, potentially influencing antipsychotic prescribing patterns. The pandemic’s impact on mental healthcare delivery, including disruption to in-person services, increased social isolation and heightened stress levels, and may have significantly altered how antipsychotics are prescribed and monitored.^15^ Although some studies investigated prescription trends during the pandemic,^16^ there is limited understanding of trends since May 2023, when COVID-19 no longer remained a pandemic-level threat.

The role of sociodemographic factors in antipsychotic prescribing trends is another area that warrants careful examination, with both ethnicity and health inequality remaining under-explored, especially in the UK context. Individuals from certain ethnic minority backgrounds, such as African and South Asian communities, are at higher risk of psychosis.^17,18^ However, early research suggests that ethnic density – that is, the proportion of individuals from the same ethnic background within a given area – may have a protective effect against psychoses.^19^ The role of ethnic density is well established in African and other South Asian communities, with higher ethnic density often associated with reduced risk of psychosis. Although research on the UK Pakistani population is limited, recent findings suggest a similar protective effect of higher ethnic density within this group.^19^ To better understand potential influences on prescribing practices, this study examines antipsychotic prescribing patterns in relation to ethnic density for the Pakistani and broader Asian and Black populations. Understanding whether prescribing patterns are influenced by ethnic density and health inequalities could provide valuable insights into treatment decisions and disparities in treatment and access to care.

Regional variations in antipsychotic prescribing add another layer of complexity to understanding antipsychotic prescribing patterns. The recent restructuring of NHS England into 7 regions and 42 integrated care boards (ICBs) presents a unique opportunity to examine these variations at a more granular level. ICBs vary in size, complexity and population characteristics (e.g. deprivation), with reports suggesting that health inequalities among ICBs are substantial and growing.^20^ ICBs are NHS statutory bodies responsible for planning and commissioning healthcare services for their local populations in England. Each ICB is responsible for the NHS budget and collaborates with local healthcare providers, such as hospitals and general practices, to develop and implement medium-term plans aligned with broader integrated care strategies.

This study aims to address knowledge gaps regarding antipsychotic prescribing in England. Specifically, we aim to:

- provide a comprehensive analysis of antipsychotic prescribing trends from April 2019 to March 2024, encompassing the pre-pandemic, pandemic and post-pandemic periods;
- quantify changes in prescribing at the national, regional and ICB levels;
- explore the association between health inequality, ethnic density and antipsychotic prescribing patterns.

## Method

### Data sources

This population-level observational study examined the prescription patterns of antipsychotic medication use across England. It utilised data from OpenPrescribing, a comprehensive database maintained by the Bennett Institute for Applied Data Science at Oxford University, and the NHS Business Service Authority (NHSBSA). This study adheres to the RECORD guidelines for reporting of observational studies (Supplementary Table 1 available at https://doi.org/10.1192/bjo.2026.10989).^21^

OpenPrescribing is an interactive database that compiles monthly records of prescriptions dispensed in primary care, and which are published by NHSBSA. It includes prescriptions written by various healthcare professionals, including doctors, pharmacists and nurses in England, and those dispensed in community settings. Prescriptions issued outside England are not included in this database. Similarly, prescriptions dispensed in hospital or prison settings are also not included in this database. The database has been widely used and cited in numerous studies.^22,23^

### Data extraction and processing

Prescription data were extracted for the period from April 2019 to March 2025. The analysis focused on ten commonly prescribed antipsychotics (FGAs: chlorpromazine, haloperidol, levomepromazine, sulpiride, flupentixol; SGAs: amisulpride, aripiprazole, olanzapine, quetiapine, risperidone. Antipsychotics were classified as FGA or SGA according to the classifications used in UK prescribing references, such as the British National Formulary. The database features an interactive search tool for each antipsychotic according to its chemical name (e.g. olanzapine) or by the British National Formulary section (e.g. section 4.2.1). Data were extracted at the ICB level, with 42 ICBs grouped into 7 NHS England regions (East of England, London, Midlands, North-East and Yorkshire, North-West, South-East and South-West) (Supplementary Table 2). The extracted data included the number of monthly prescription items for each ICB over the study period, with a prescription item corresponding to a single prescription for a particular drug.

To provide additional context and enable deeper analysis, health inequality indices were incorporated; these indices were extracted from documents published by NHS England.^24^ The index values are relative to a baseline of 1 (England average). An index <1 indicates lower health inequalities, with >1 indicating higher inequalities, for a given ICB.

Population data for each ICB were obtained from the Waterfalls analysis,^25^ which NHS England uses as part of the financial allocation process. ICB-level ethnicity data were obtained from NHS Digital’s combined Hospital Episode Statistics and COVID-19 planning data.^26^ These documents are publicly available on the NHS website, and further details about this can be found on the NHS website.^26^ We calculated ethnic density for each ICB by dividing the number of residents from a specific ethnic group (Pakistani, broader Asian or Black) by the total ICB population, generating a percentage measure where higher values indicate a higher relative proportion of that ethnic group.^19^

### Statistical analyses

Monthly prescription data for all antipsychotics were summed to calculate the total number of prescription items per year, from April 2019 to March 2025. The number of prescriptions for each antipsychotic was first calculated at the ICB level and then aggregated by region to obtain the total prescription count for each region. The regional data were further aggregated to provide the national-level prescription count for all antipsychotics. Linear regression analysis was performed to identify trends in antipsychotic use over the study period. The mean annual change was calculated by dividing the regression coefficient by the baseline prescription count (2019). Statistical significance was determined using a 95% confidence interval and a *P*-value threshold of 0.05.

The influence of ethnic density and health inequalities on the use of antipsychotic medications was explored using a generalised additive model, which allows for a non-linear, complex association by using smooth functions. The model treated health inequalities as a smooth term due to their potential non-linear relationship with antipsychotic prescription rates. The relationships of these terms with FGAs and SGAs were also examined separately to identify differences in their impact on prescription counts. Linearity was assessed by visual inspection of the residual plot. The smooth terms were modelled using penalised regression splines to estimate their non-linear effects on prescription rates. All variables were treated as continuous variables. The significance of smooth terms was assessed using the *F*-test. The overall model fit was evaluated using an adjusted *R* ^2^, generalised cross-validation score, Akaike information criterion and Bayesian information criterion. All analyses were conducted using Microsoft Excel, version 2511 for Windows (Microsoft Corporation, Washington, USA; https://www.microsoft.com/en-gb/microsoft-365/excel) and R, version 4.5.2 for Windows (R Foundation for Statistical Computing, Vienna, Austria; https://www.R-project.org/).

## Results

Overall, the use of antipsychotics significantly increased, from 185.55 to 199.85 prescriptions per 1000 population (*P* < 0.01), from April 2019 to March 2025. The use of SGAs also increased significantly, from 168.48 to 186.27 prescriptions per 1000 population (*P* < 0.01). By contrast, the use of FGAs declined from 17.08 to 13.97 prescriptions per 1000 population during the same period (*P* < 0.01) (Table 1).

**Table 1:** National prescription count per 1000 population of first- and second-generation antipsychotics

Drugs	Prescription count per 1000 population						Regression output				
	2019	2020	2021	2022	2023	2024	Estimate	95% CI lower	95% CI upper	s.e.	*P*-value
First-generation antipsychotics											
Chlorpromazine	4.46	4.20	3.92	3.58	3.24	3.00	−0.30	−0.32	−0.29	0.01	<0.01
Haloperidol	5.90	6.10	5.72	5.62	5.42	5.31	−0.15	−0.21	−0.09	0.03	0.01
Levomepromazine	2.38	2.89	2.63	2.52	2.66	2.62	0.01	−0.08	0.10	0.04	0.81
Sulpiride	2.20	1.84	1.69	1.59	1.51	1.41	−0.14	−0.19	−0.10	0.02	<0.01
Flupentixol	2.14	1.75	1.30	1.27	1.25	1.24	−0.17	−0.27	−0.07	0.05	0.03
Total	17.08	16.83	15.26	14.58	13.97	13.58	−0.77	−0.93	−0.60	0.08	<0.01
Second-generation antipsychotics											
Amisulpride	7.04	7.08	6.91	6.78	6.52	6.41	−0.14	−0.18	−0.10	0.02	<0.01
Aripiprazole	21.65	23.55	25.49	26.64	27.93	29.72	1.56	1.43	1.70	0.07	<0.01
Olanzapine	43.30	44.29	44.66	44.46	44.33	44.23	0.13	−0.08	0.34	0.11	0.29
Quetiapine	65.66	69.93	72.90	74.14	75.68	76.18	2.03	1.38	2.68	0.33	<0.01
Risperidone	30.65	30.77	30.48	30.28	29.98	29.73	−0.20	−0.27	−0.14	0.03	<0.01
Total	168.48	176.31	180.44	182.17	182.92	186.27	3.16	2.04	4.27	0.57	0.01
Overall antipsychotics	185.55	193.14	195.70	196.75	196.89	199.85	2.39	1.29	3.50	0.56	<0.01

**Fig. 1:**
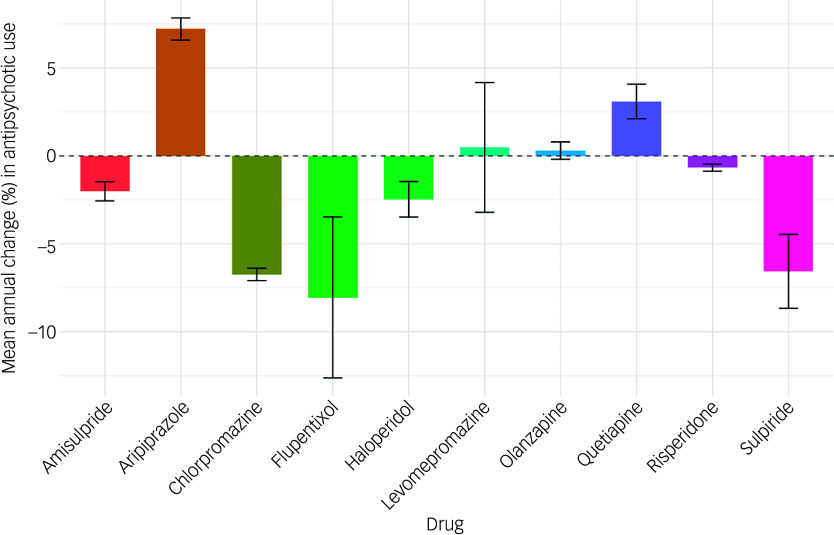
Mean annual change (%) in antipsychotic prescription items, with 95% confidence intervals.

Among all antipsychotics, quetiapine remained the most prescribed throughout the study period (3.69−4.46 million prescriptions), followed by olanzapine (2.43−2.59 million prescriptions) and risperidone (1.72−1.74 million prescriptions) (Supplementary Fig. 1). However, the rates of change varied considerably across different antipsychotics. Aripiprazole showed the highest annual increase, of 7.21% (95% CI = 6.58%, 7.84%), followed by quetiapine at 3.09% (95% CI = 2.11%, 4.08%). Interestingly, amisulpride (−2.01%, 95% CI = −2.56%, −1.47%) and risperidone (−0.67%, 95% CI = −0.87%, −0.47%) were the two SGAs showing an overall decrease in use, whereas levomepromazine was the only FGA to show an overall annual increase, of 0.48% (95% CI = −3.21%, 4.17%). By contrast, the use of all other SGAs increased and the use of all other FGAs decreased. Flupentixol showed the steepest drop (−8.04%, 95% CI = −12.62%, −3.47%), followed by chlorpromazine (−6.73%, 95% CI = −7.09%, −6.39%) and sulpiride (−6.56%, 95% CI = −8.66%, −4.46%) (Fig. 1).

Regional analysis showed marked disparities in antipsychotic use across England. The North-West region consistently exhibited the highest prescription rates, ranging from 220.47 to 227.89 prescriptions per 1000 population from 2019 to 2022, before dropping to 219.76 in 2024. However, the London region demonstrated the most marked shift, with prescription rates surging from 182.0 to 209.8 per 1000 population, overtaking regions including the North-East and Yorkshire (206.57 to 213.91 per 1000 population). Prescription counts across different regions of England are summarised in Table 2.

**Table 2:** Regional prescription count per 1000 population of first- and second-generation antipsychotics

Region	Prescription count per 1000 population																	
	First-generation antipsychotics						Second-generation antipsychotics						Total antipsychotics					
	2019	2020	2021	2022	2023	2024	2019	2020	2021	2022	2023	2024	2019	2020	2021	2022	2023	2024
East of England	13.98	13.68	12.44	11.98	11.31	11.07	164.91	174.22	178.73	183.05	184.27	187.08	178.89	187.90	191.17	195.02	195.58	198.15
London	14.31	14.11	13.10	12.92	12.82	12.87	167.69	176.30	182.67	190.49	195.76	205.69	182.00	190.41	195.78	203.41	208.58	218.56
Midlands	16.85	16.35	14.89	14.49	13.81	13.30	156.01	162.34	167.75	171.45	172.16	174.44	172.85	178.69	182.64	185.94	185.97	187.74
North-East and Yorkshire	21.67	20.95	18.81	18.07	17.16	16.78	184.90	188.43	191.39	194.37	194.16	197.13	206.57	209.39	210.20	212.44	211.32	213.91
North-West	23.34	22.92	21.19	20.21	18.83	17.66	197.14	201.34	204.88	207.67	203.23	202.11	220.47	224.26	226.08	227.89	222.06	219.76
South-East	11.89	12.00	10.55	10.29	10.16	10.09	151.80	158.88	162.98	164.91	166.44	170.77	163.69	170.88	173.53	175.20	176.60	180.86
South-West	18.57	17.66	15.77	14.84	14.36	13.81	160.20	164.56	165.91	166.02	165.91	166.36	178.78	182.22	181.68	180.86	180.27	180.17

Region	Regression output														
	First-generation antipsychotics					Second-generation antipsychotics					Total antipsychotics				
	Estimate	95% LCI	95% UCI	s.e.	*P*-value	Estimate	95% LCI	95% UCI	s.e.	*P*-value	Estimate	95% LCI	95% UCI	s.e.	*P*-value
East of England	−0.63	−0.76	−0.51	0.06	<0.01	4.15	2.90	5.41	0.64	<0.01	3.52	2.29	4.75	0.63	<0.01
London	−0.32	−0.48	−0.16	0.08	0.02	7.32	6.81	7.83	0.26	<0.01	7.00	6.42	7.58	0.30	<0.01
Midlands	−0.74	−0.87	−0.60	0.07	<0.01	3.58	2.54	4.62	0.53	<0.01	2.85	1.88	3.81	0.49	<0.01
North-East and Yorkshire	−1.04	−1.28	−0.81	0.12	<0.01	2.32	1.79	2.86	0.27	<0.01	1.28	0.80	1.75	0.24	0.01
North-West	−1.19	−1.33	−1.05	0.07	<0.01	0.95	−0.65	2.56	0.82	0.31	−0.24	−1.91	1.43	0.85	0.79
South-East	−0.42	−0.62	−0.22	0.10	0.01	3.41	2.55	4.28	0.44	<0.01	2.99	2.14	3.84	0.43	<0.01
South-West	−0.99	−1.22	−0.76	0.12	<0.01	1.00	0.25	1.74	0.38	0.06	−0.37	−1.29	0.56	1.35	0.36

95% LCI, 95% CI lower; 95% UCI, 95% CI upper.

**Fig. 2:**
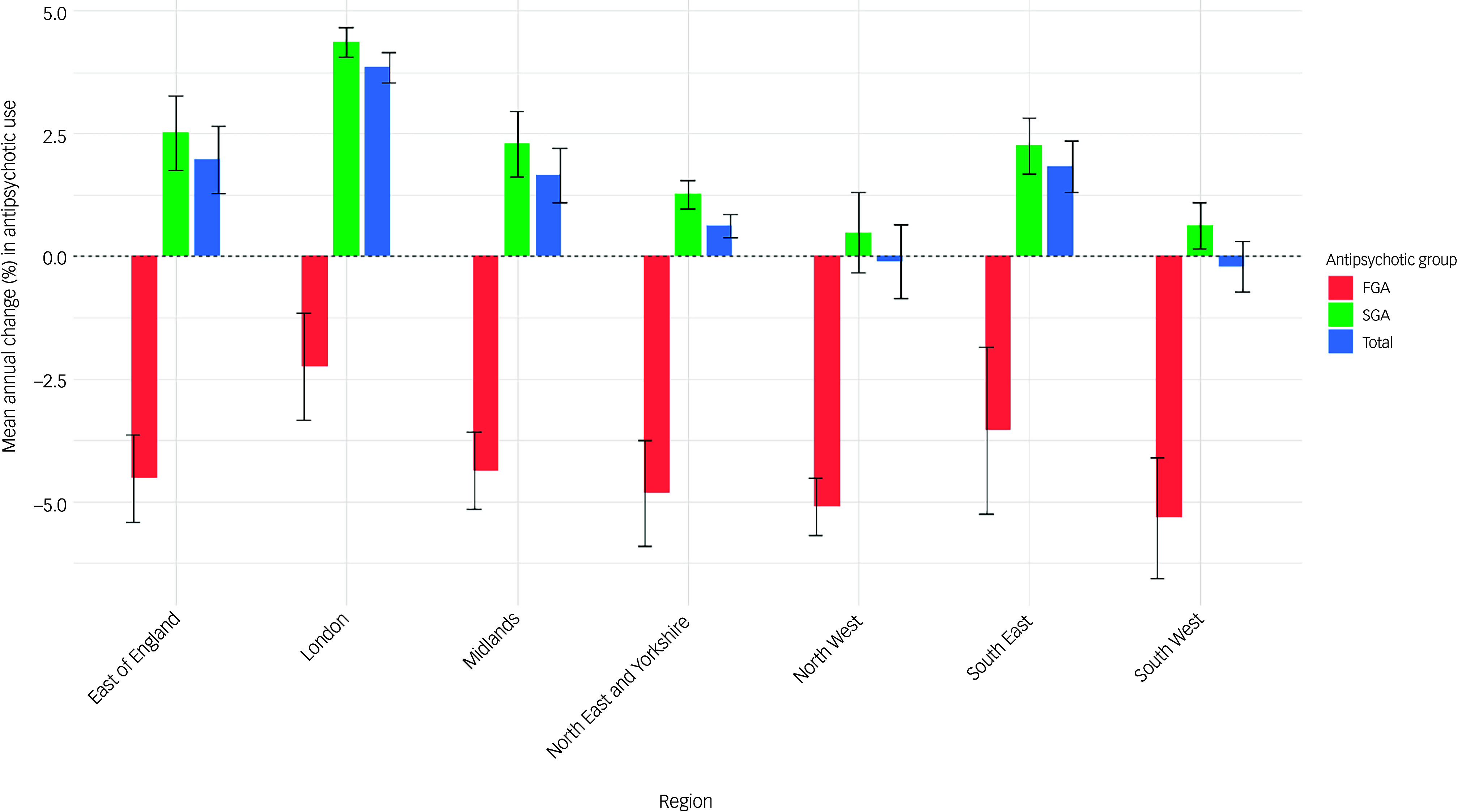
Mean percentage change in first-generation (FGA), second-generation (SGA) and total antipsychotic items with 95% confidence interval, by region.

The mean annual change in prescriptions varied significantly across regions, with the London region showing the highest annual increase, at 3.85% (95% CI = 3.53%, 4.16%), whereas the South-West region experienced a decrease of −0.21% (95% CI = −0.72%, 0.31%). Similar trends were noted for SGAs, with London exhibiting the highest increase (4.37%, 95% CI = 4.06%, 4.67%) and North-West the lowest (0.48%, 95% CI = −0.33%, 1.30%). By contrast, decline in the use of FGAs was highest in the South-West (−5.33%, 95% CI = −6.56%, −4.10%) and lowest in London (−2.25%, 95% CI = −3.34%, −1.15%) (Fig. 2).

**Fig. 3:**
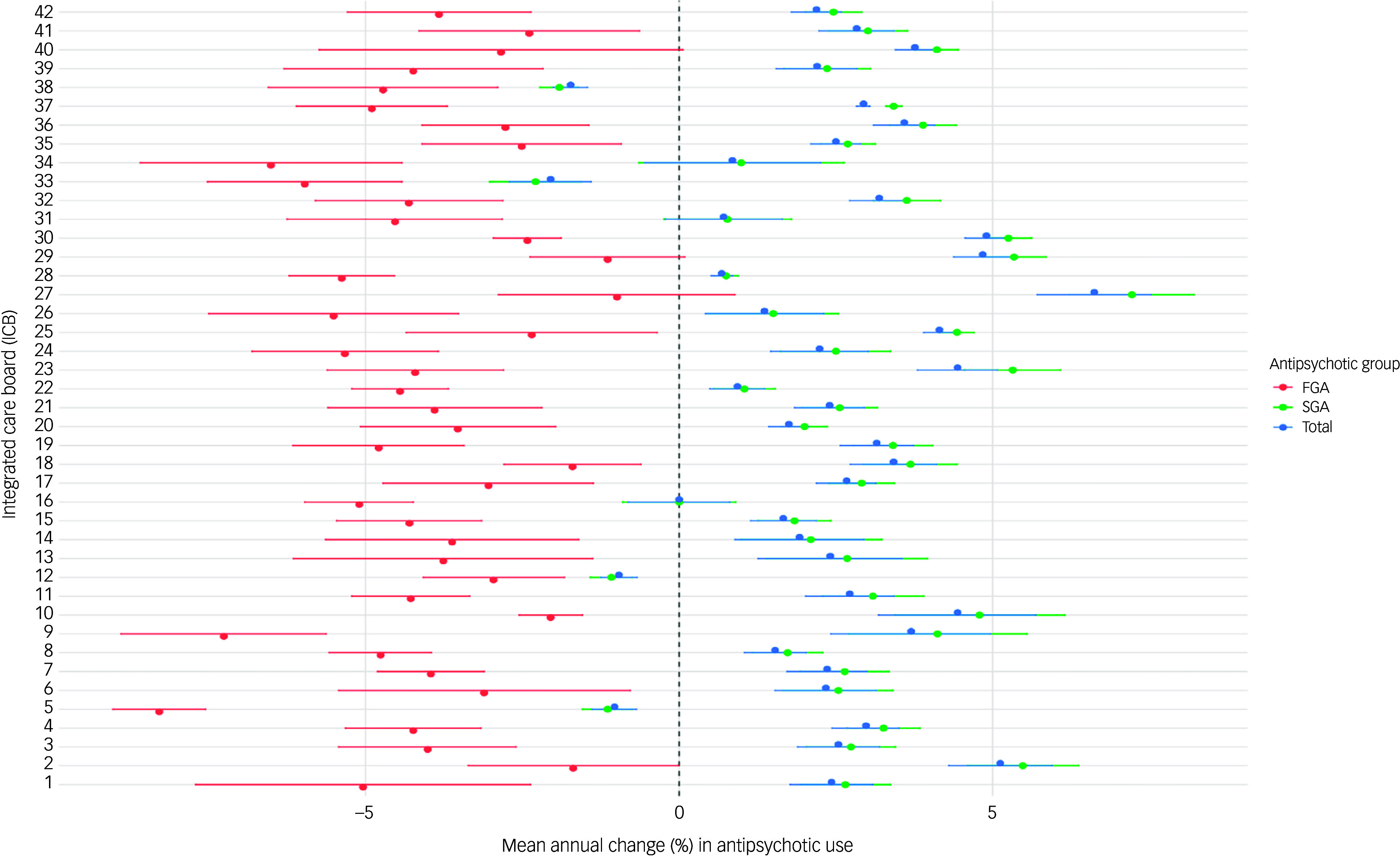
Mean percentage change in first-generation (FGA), second-generation (SGA) and total antipsychotic items with 95% confidence intervals, by integrated care board (ICB). 1. NHS Bath and North-East Somerset, Swindon and Wiltshire ICB. 2. NHS Bedfordshire, Luton and Milton Keynes ICB. 3. NHS Birmingham and Solihull ICB. 4. NHS Black Country ICB. 5. NHS Bristol, North Somerset and South Gloucestershire ICB. 6. NHS Buckinghamshire, Oxfordshire and Berkshire West ICB. 7. NHS Cambridgeshire and Peterborough ICB. 8. NHS Cheshire and Merseyside ICB. 9. NHS Cornwall and the Isles of Scilly ICB. 10. NHS Coventry and Warwickshire ICB. 11. NHS Derby and Derbyshire ICB. 12. NHS Devon ICB. 13. NHS Dorset ICB. 14. NHS Frimley ICB. 15. NHS Gloucestershire ICB. 16. NHS Greater Manchester ICB. 17. NHS Hampshire and Isle of Wight ICB. 18. NHS Herefordshire and Worcestershire ICB. 19. NHS Hertfordshire and West Essex ICB. 20. NHS Humber and North Yorkshire ICB. 21. NHS Kent and Medway ICB. 22. NHS Lancashire and South Cumbria ICB. 23. NHS Leicester, Leicestershire and Rutland ICB. 24. NHS Lincolnshire ICB. 25. NHS Mid- and South Essex ICB. 26. NHS Norfolk and Waveney ICB. 27. NHS North Central London ICB. 28. NHS North-East and North Cumbria ICB. 29. NHS North-East London ICB. 30. NHS North-West London ICB. 31. NHS Northamptonshire ICB. 32. NHS Nottingham and Nottinghamshire ICB. 33. NHS Shropshire, Telford and the Wrekin ICB. 34. NHS Somerset ICB. 35. NHS South-East London ICB. 36. NHS South-West London ICB. 37. NHS South Yorkshire ICB. 38. NHS Staffordshire and Stoke-on-Trent ICB. 39. NHS Suffolk and North-East Essex ICB. 40. NHS Surrey Heartlands ICB. 41. NHS Sussex ICB. 42. NHS West Yorkshire ICB. NHS, National Health Service.

At the ICB level, even more marked variations were observed. Population-adjusted prescription count was highest in Norfolk and Waveney (242 prescriptions per 1000 population in 2024) and lowest in Staffordshire and Stoke-on-Trent (118.61 prescriptions per 1000 population in 2024) (Supplementary Table 3). Annual changes in prescription rates ranged from 6.62% (95% CI = 5.71%, 7.53%) in North Central London to −2.05% (95% CI = −2.71%, −1.40%) in Shropshire, Telford and the Wrekin. A decline in the use of antipsychotics was also noted in Staffordshire and Stoke-on-Trent (−1.73%, 95% CI = −2.01%, −1.46%), Bristol, North Somerset and South Gloucestershire (−1.03%, 95% CI = −1.39%, −0.68%) and Devon (−0.96%, 95% CI = −1.25%, −0.67%). Population-adjusted ICB-level variation in FGA, SGA and total antipsychotics is presented in Fig. 3.

The use of SGAs also varied widely across ICBs, with North Central London showing the highest annual increase (7.22%, 95% CI = 6.23%, 8.21%) and Shropshire, Telford and the Wrekin showing the highest decrease (−3.02%, 95% CI = −1.56%, −2.05%). By contrast, mean change in the use of FGAs was highest in Bristol, North Somerset and South Gloucestershire (−8.29%, 95% CI = −9.03%, −7.55%) and lowest in North Central London (−0.99%, 95% CI = −2.88%, 0.89%) (Fig. 3).

Further analysis of specific SGAs revealed substantial variations across ICBs. The mean change in the use of amisulpride ranged between −9.52% (95% CI = −13.22%, −5.82%) and 4.63% (95% CI = 2.15%, 7.10%); of aripiprazole between 1.87% (95% CI = 0.95%, 2.79%) and 17.99% (95% CI = 15.28%, 20.70%); and of quetiapine between −1.41% (95% CI = −2.88%, −0.6%) and 9.98% (95% CI = 7.73%, 12.23%). Olanzapine and risperidone showed relatively less variation compared with other SGAs, ranging between −3.72% (95% CI = −4.71%, −2.73%) and 5.13% (95% CI = 4.14%, 6.13%), and −4.50% (95% CI = −5.81%, −3.18%) and 4.86% (95% CI = 2.92%, 6.81%), respectively (Supplementary Fig. 2).

**Fig. 4:**
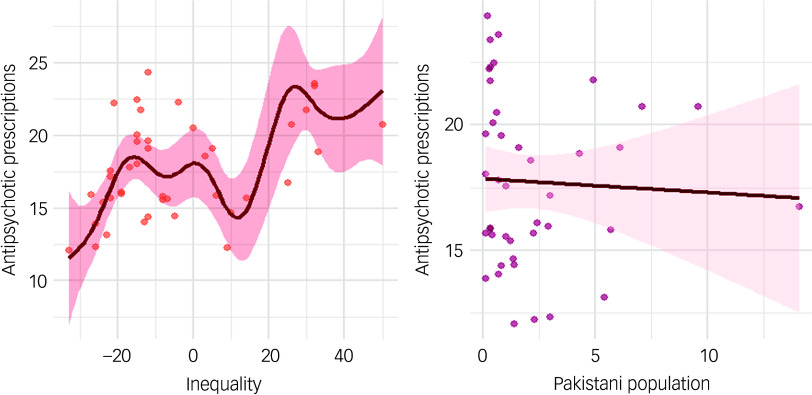
Association between total antipsychotic prescriptions and health inequalities and ethnicity (UK Pakistani).

The generalised additive model showed a significant non-linear relationship between prescription count and health inequalities. For total prescription count, health inequalities (estimated degrees of freedom (EDF) 7.78, *P* < 0.01) exhibited a complex, non-linear relationship, whereas higher Pakistani ethnic density was associated with a general decline in prescriptions (EDF −0.5, *P* = 0.02) (Table 3 and Fig. 4). The model analysis of FGA prescriptions showed a significant linear association with both health inequality (EDF 1.00, *P* < 0.01) and Pakistani ethnic density (EDF −0.04, *P* < 0.01) (Supplementary Table 4 and Supplementary Fig. 3). By contrast, the association between SGA prescriptions and health inequality was non-linear (EDF 7.74, *P* < 0.01), whereas prescribing rates decreased with increasing Pakistani ethnic density (Supplementary Table 5 and Supplementary Fig. 4). In contrast to the Pakistani population, no significant associations were found for the broader Asian population (EDF −0.11, *P* = 0.06) or Black population (EDF −18.92, *P* = 0.11) when examined in separate models (Supplementary Table 6a,b and Supplementary Fig. 5a,b).

**Table 3:** Regression and smooth-term analysis of total prescription count with ethnicity (Pakistani) and health inequality

Variables	Estimate	s.e.	*t*-value	*P*-value
Intercept	18.82	0.59	31.96	<0.01
Ethnicity (Pakistani)	−0.5	0.2	−2.45	0.02

	EDF	Ref. EDF	*F*-value	*P*-value
Health inequality	7.78	8.6	4.83	<0.01

EDF, estimated degrees of freedom; Ref. EDF, reference estimated degrees of freedom. *R* ^2^ (adjusted) 0.46; deviation explained 57.9; generalised cross validation 7.87; scale estimate 6.04.

## Discussion

This study aimed to examine antipsychotic prescription trends in England covering the periods before, during and after the COVID-19 pandemic at national, regional and ICB levels. The study sought to examine potential disparities in antipsychotic prescription rates across the country and their association with health inequality and ethnic density. The study found significant shifts in antipsychotic prescription practices, characterised by an overall increase in prescriptions and with a notable rise in SGAs and a decline in FGAs. The study found substantial variation at the regional and ICB levels, with some areas exhibiting consistently higher prescription rates and others experiencing more pronounced changes over time, suggesting that localised factors may influence prescribing patterns. Furthermore, the study found a significant association between antipsychotic prescription rates and both health inequality and Pakistani ethnic density. It highlights the potential for targeted interventions and further research to ensure the equitable and appropriate use of antipsychotics across England.

The findings of this study align with global trends observed in regard to antipsychotic use. A comprehensive review of 88 empirical studies from 2002 to 2023 reported an absolute rise in SGAs of up to 50%.^8^ However, it is important to note that timelines for the launch of different SGAs varied across different regions, which may have influenced regional prescribing trends across different settings. Similar trends have been observed in country-specific studies across the USA,^27^ Australia,^28^ Europe,^29^ Japan^30^ and other Asian countries.^31^ In the UK, a study analysing prescription trends between 2007 and 2014 found a consistent increase in antipsychotic prescriptions, with SGAs accounting for 79.9% of the total.^14^ Another study investigating antipsychotic prescribing in children and adolescents reported an average increase of 3.3% per year (2000–2019^9^). These findings are supported by additional analyses of national prescribing data, which together provide a comprehensive overview of prescribing patterns across different populations and time periods.^10–13^ Although these studies provide valuable context, the findings of the current study offer a timely and nuanced examination of recent trends in England, including the factors driving increased antipsychotic use.

The increase in antipsychotic use and preferential prescribing of SGAs can be attributed to several factors. Antipsychotics are used to treat a wide range of mental disorders beyond their original indication for schizophrenia, including bipolar disorder, severe depression, anxiety, dementia, learning disorders and other off-label conditions.^32^ Because both the awareness and diagnosis of these conditions have increased over time, the use of antipsychotics has expanded accordingly. Furthermore, changes in the antipsychotic market and prescribing environment may have contributed to the observed trends. For example, the relative increase in prescribing of newer SGAs such as aripiprazole may reflect changes in guideline recommendations, potential drug promotional activities and local formulary positioning.

The COVID-19 pandemic has been linked to rising incidence of mental health issues in England^33^ and worldwide,^34^ which may have contributed to the increased use of antipsychotics in the past 5 years. However, pandemic-related disruptions to routine clinical review may also have contributed. Reduced face-to-face contact and service capacity may have limited opportunities for medication review and de-prescribing, potentially resulting in antipsychotics being initiated for new patients whereas existing prescriptions were continued for longer than usual.

Other studies have also suggested a strong association between the pandemic and the use of antipsychotics. For example, a multinational study reported a marked increase in antipsychotic prescribing in the USA, the UK, France, Italy and South Korea.^16^ In England, one study reported that the use of antipsychotics in dementia increased from 82.75 per 1000 patients in 2019 to 90.1 per 1000 patients in 2021,^35^ complementing other studies showing similar findings.^36^ Our findings corroborate these observations, showing the impact of the pandemic on antipsychotic prescriptions beyond the acute phase. This prolonged impact underscores the need for ongoing monitoring and adaptive strategies in mental healthcare delivery.

Evidence regarding the relative effectiveness of SGAs compared with FGAs is mixed.^37,38^ One meta-analysis indicated that, although some SGAs (e.g. olanzapine, clozapine) are more efficacious than FGAs for certain symptomatic outcomes, overall differences are small and heterogeneous.^37^ Large clinical trials, such as the US Clinical Antipsychotic Trials of Intervention Effectiveness and the UK CUtLASS study, found no consistent superiority of SGAs over FGAs in terms of symptom improvement, quality of life or adverse effects.^5,39^ Accordingly, national guidelines in the UK, such as those of the National Institute for Health and Care Excellence, do not recommend a specific antipsychotic choice for conditions such as schizophrenia. Clinicians’ decisions may instead be influenced by perceived safety, tolerability, sedative or mood-modulating effects, and the broader context of each patient’s condition. Further research is needed to better understand how clinicians and patients prioritise SGAs versus FGAs in routine practice.

The substantial variations in antipsychotic prescribing patterns observed at both regional and ICB levels underscore the complex interplay of local factors influencing mental healthcare delivery. These variations align with previous studies that have identified geographical variations in mental healthcare across England.^40,41^ Possible reasons for such variations may reflect differences in local population needs, healthcare resource allocations or local prescribing cultures. Regional policies, formularies and key opinion leaders can also influence the preferred antipsychotics within a given area. In addition, system-level factors such as guideline interpretation, clinician preference or increased visibility of certain drugs through potential promotional activity may also contribute to the observed differences. Furthermore, the availability of non-pharmacological interventions and variations in specialist mental health services may explain the variations in the use of antipsychotics across England, as evidenced by the significant disparities in wait times for talking therapy across different regions.^3^ These findings emphasise the need for a more granular analysis of local prescribing determinants, and raise important questions about health equity and the standardisation of care across different geographical areas in England.

The distinct characteristics of populations in these areas can partly explain variations at the regional and ICB levels. The analysis of ethnicity and health inequality in this study provides crucial insights into these local differences, further clarifying the drivers behind antipsychotic prescribing trends. The strong association between inequality indices and antipsychotic use suggests that areas with higher levels of health disparity may face greater mental health challenges, potentially leading to an increase in antipsychotic use. The inverse association between Pakistani ethnic density and antipsychotic prescribing rates aligns with the concept of ethnic density, which suggests that individuals from ethnic minority backgrounds residing in areas with higher proportions of their own ethnic group may benefit from protective social effects. Prior research has shown that higher ethnic density is linked to better mental health outcomes, reduced stress from discrimination and greater social cohesion, which may collectively reduce the perceived need for antipsychotic treatment.^42^ However, an alternative interpretation is that lower prescribing rates in areas with higher Pakistani ethnic density may reflect barriers to healthcare access rather than a true reduction in clinical need. Cultural differences in treatment-seeking behaviour, medical distrust and communication barriers between patients and clinicians may contribute to disparities in prescribing patterns.^43^ In addition, service-level factors, such as staff recruitment and retention challenges or broader resource constraints in deprived areas, may also influence prescribing patterns. Furthermore, prescribing choices may be influenced by differences in treatment preferences, socioeconomic status or variations in clinical prescribing behaviour. Further research is needed to confirm and establish whether the observed patterns reflect a genuine protective effect of high ethnic density or an unmet treatment need due to systemic inequalities in healthcare provision.

### Strengths and limitations

The primary strength of this study lies in it being the first comprehensive examination of antipsychotic prescribing trends covering a significant period before, during and after the COVID-19 pandemic. Additionally, this study examined trends at the national, regional and ICB levels, providing deeper insights into patterns across different geographical areas. This multi-tiered approach provides valuable information on potential disparities in antipsychotic use.

This study has certain limitations. It used aggregated data, which do not reflect individual patient-level associations between sociodemographic factors and antipsychotic use. The data-set included prescriptions dispensed in community settings in England but excluded medications dispensed or administered in hospitals and other settings (e.g. depot antipsychotics). The study included only the five most commonly prescribed antipsychotics in each class, and may not account for all antipsychotics and factors influencing their prescribing, such as physician preferences, local hospital policies or changes in diagnostic practices during the pandemic. Furthermore, the FGA/SGA classification is not a perfect construct and may oversimplify pharmacological differences, particularly for agents such as sulpiride and amisulpride. Because prescribing patterns are complex and are influenced by multiple factors, the variables used in this study may not fully account for the observed trends. Although age and gender standardisation was not applied in this analysis, differences across ICBs are modest and are unlikely to substantially affect the observed national trends. Although the study provides valuable insights into prescribing trends, it cannot directly assess the appropriateness of prescriptions, the potential impact of pharmaceutical promotion, medication adherence or clinical outcomes.

### Implications

This study has several clinical, research and policy implications. The growing use of antipsychotics, particularly SGAs, underscores the need for clinicians to remain vigilant about the associated metabolic and cardiovascular risks. Proactive strategies should be implemented to monitor and mitigate these side-effects in individuals. Further research is needed to determine the most effective management of these side-effects. Furthermore, variations in prescribing patterns at the regional and ICB levels highlight the need to standardise clinical practices to address unwarranted disparities in service provision. At the same time, it is crucial to account for socioeconomic and cultural factors when assessing and addressing mental health needs. By adopting evidence-based standardisation, we can reduce unjustified variation in care while preserving the flexibility needed to deliver person-centred, tailored care.

Future research should investigate the drivers of disparities in antipsychotic prescribing, including patient-, provider- and systemic-level factors. Additionally, there is a need for longitudinal studies to explore the long-term impact of COVID-19 on antipsychotic use. From a policy perspective, these findings highlight the need for the development of culturally sensitive interventions to address socioeconomic disparities in mental healthcare, along with policies that ensure equitable access to, and the judicious use of, antipsychotics across England.

Overall, this study reveals a significant shift in antipsychotic prescribing patterns in England between April 2019 and March 2025, marked by a substantial increase in SGAs and a decrease in FGAs. These changes reflect the complex interplay of socioeconomic and regional factors, including the COVID-19 pandemic’s profound impact on mental health and shifts in antipsychotic use. The notable variations observed at the regional and ICB levels, along with the associations between ethnic density and antipsychotic use, suggest the protective effect of higher ethnic density. These findings highlight the need for a tailored approach to mental healthcare delivery, one that integrates evidence-based clinical practices while also accounting for local demographic and cultural needs. Furthermore, standardisation of care where appropriate, combined with this tailored approach, will be essential for reducing disparities and improving outcomes in mental healthcare.

## Supporting information

Khan et al. supplementary material Khan et al. supplementary material

## Data Availability

Data are available in a public, open access repository. All data used in this study are publicly available. The data supporting this study’s findings are available from the NHS Business Services Authority (NHSBSA) (https://www.nhsbsa.nhs.uk).

## Supplementary material

The supplementary material is available online at https://doi.org/10.1192/bjo.2026.10989
